# Vaccination Coverage by Age 24 Months Among Children Born in 2016 and 2017 — National Immunization Survey-Child, United States, 2017–2019

**DOI:** 10.15585/mmwr.mm6942a1

**Published:** 2020-10-23

**Authors:** Holly A. Hill, David Yankey, Laurie D. Elam-Evans, James A. Singleton, S. Cassandra Pingali, Tammy A. Santibanez

**Affiliations:** 1 Immunization Services Division, National Center for Immunization and Respiratory Diseases, CDC.

Immunization has been described as a “global health and development success story,” and worldwide is estimated to prevent 2–3 million deaths annually.* In the United States, the Advisory Committee on Immunization Practices (ACIP) currently recommends vaccination against 14 potentially serious illnesses by the time a child reaches age 24 months (*1*). CDC monitors coverage with ACIP-recommended vaccines through the National Immunization Survey-Child (NIS-Child); data from the survey were used to estimate vaccination coverage at the national, regional, state, territorial, and selected local area levels^†^ among children born in 2016 and 2017. National coverage by age 24 months was ≥90% for ≥3 doses of poliovirus vaccine, ≥3 doses of hepatitis B vaccine (HepB), and ≥1 dose of varicella vaccine (VAR); national coverage was ≥90% for ≥1 dose of measles, mumps, and rubella vaccine (MMR), although MMR coverage was <90% in 14 states. Coverage with ≥2 doses of influenza vaccine was higher for children born during 2016–2017 (58.1%) than for those born during 2014–2015 (53.8%) but was the lowest among all vaccines studied. Only 1.2% of children had received no vaccinations by age 24 months. Vaccination coverage among children enrolled in Medicaid or with no health insurance was lower than that among children who were privately insured. The prevalence of being completely unvaccinated was highest among uninsured children (4.1%), lower among those enrolled in Medicaid (1.3%), and lowest among those with private insurance (0.8%). The largest disparities on the basis of health insurance status occurred for ≥2 doses of influenza vaccine and for completion of the rotavirus vaccination series. Considering the disruptions to health care provider operations caused by the coronavirus disease 2019 (COVID-19) pandemic, extra effort will be required to achieve and maintain high levels of coverage with routine childhood vaccinations. Providers, health care entities, and public health authorities can communicate with families about how children can be vaccinated safely during the pandemic, remind parents of vaccinations that are due for their children, and provide all recommended vaccinations to children during clinic visits. This will be especially important for 2020–21 seasonal influenza vaccination to mitigate the effect of two potentially serious respiratory viruses circulating in the community simultaneously.

The NIS-Child is conducted annually as a random-digit–dialed telephone survey^§^ of parents and guardians of children aged 19–35 months. Sociodemographic information is collected during the telephone interview, and the respondent is asked to identify all providers who administered vaccines to the child. When consent is obtained, a survey is mailed to each provider requesting the child’s vaccination history. If survey responses from multiple providers are returned for a given child, the information is synthesized into a single, comprehensive vaccination history, which is then used to calculate vaccination coverage estimates. NIS-Child data from survey years 2017–2019 were combined to identify 25,970 children with adequate provider data^¶^ who were born in 2016 and 2017. For survey year 2019, the household response rate** was 21.1%, and 49.4% of children with completed household interviews had adequate provider data. Kaplan-Meier (time to event) analysis was used to estimate vaccination coverage for most vaccines by age 24 months while still using information from children whose vaccination status was assessed at age 19–23 months. The birth dose of HepB was assessed at age 3 days, and the rotavirus series was assessed at age 8 months to correspond with timing of ACIP recommendations for those vaccines. Coverage with ≥2 doses of hepatitis A vaccine (HepA) was estimated by age 35 months (the maximum age included in the survey) using Kaplan-Meier methods, because the second HepA dose can be administered as late as 41 months under the current recommended immunization schedule. Coverage estimates for children born in 2016 and 2017 were compared with corresponding estimates for children born in 2014 and 2015. Estimates for children born in 2014 and 2015 were calculated using NIS-Child data from 2015–2018. Differences were evaluated using t-tests on weighted data; p-values <0.05 were considered statistically significant. Analyses were performed using SAS (version 9.4; SAS Institute) and SUDAAN (version 11; Research Triangle Institute).

## National Vaccination Coverage

Among children born in 2016 and 2017, the percentage with up to date coverage by age 24 months was highest for ≥3 doses of poliovirus vaccine (92.1%), ≥3 doses of HepB (91.4%), ≥1 dose of MMR (90.7%), and ≥1 dose of VAR (90.0%) (Table 1). Compared with children born in 2014 and 2015, coverage increased for ≥2 doses of influenza vaccine (4.3 percentage points), the HepB birth dose (4.2 percentage points), completion of the rotavirus vaccination series (3.2 percentage points), the combined 7-vaccine series^††^ (2.1 percentage points), and ≥1 dose of HepA (1.8 percentage points). However, coverage remained lowest for ≥2 doses of influenza vaccine (58.1%), the combined 7-vaccine series (70.5%), completion of the rotavirus vaccination series (75.3%), and the HepB birth dose (76.3%). The proportion of children who received no vaccinations by age 24 months was 1.2%.

**TABLE 1:** Estimated vaccination coverage by age 24 months,* among children born during 2014–2017 for selected vaccines and doses — National Immunization Survey-Child, United States, 2015–2019

Vaccine/Dose	Birth years^†^		Difference (2016–2017) to (2014–2015)
	2014–2015	2016–2017	
	% (95% CI)	% (95% CI)	% (95% CI)
**DTaP^§^**			
≥3 doses	93.4 (92.9 to 94.0)	93.3 (92.5 to 94.0)	−0.2 (−1.1 to 0.7)
≥4 doses	80.4 (79.4 to 81.3)	80.6 (79.4 to 81.8)	0.2 (−1.3 to 1.7)
**Poliovirus (≥3 doses)**	91.8 (91.1 to 92.5)	92.1 (91.4 to 92.9)	0.3 (−0.7 to 1.4)
**MMR (≥1 dose)^¶^**	90.3 (89.6 to 90.9)	90.7 (89.8 to 91.5)	0.4 (−0.7 to 1.5)
Hib**			
Primary series	92.3 (91.7 to 93.0)	92.2 (91.3 to 93.0)	−0.1 (−1.2 to 0.9)
Full series	79.6 (78.6 to 80.6)	79.9 (78.6 to 81.1)	0.3 (−1.3 to 1.8)
**HepB**			
Birth dose^††^	72.1 (70.9 to 73.3)	76.3 (75.0 to 77.5)	4.2 (2.5 to 5.9)^§§^
≥3 doses	90.4 (89.6 to 91.1)	91.4 (90.5 to 92.2)	1.0 (−0.1 to 2.1)
**VAR (≥1 dose)^¶^**	89.7 (88.9 to 90.3)	90.0 (89.1 to 90.9)	0.4 (−0.8 to 1.5)
**PCV**			
≥3 doses	91.5 (90.8 to 92.2)	91.6 (90.8 to 92.4)	0.1 (−0.9 to 1.2)
≥4 doses	81.2 (80.2 to 82.1)	81.7 (80.5 to 82.8)	0.5 (−1.0 to 2.0)
**HepA**			
≥1 dose	84.0 (83.1 to 84.8)	85.8 (84.7 to 86.8)	1.8 (0.5 to 3.2)^§§^
≥2 doses (by age 35 mos)	74.9 (73.5 to 76.3)	76.9 (75.2 to 78.5)	2.0 (−0.2 to 4.1)
**Rotavirus** (by age 8 mos)^¶¶^	72.2 (71.0 to 73.3)	75.3 (74.1 to 76.5)	3.2 (1.5 to 4.8)^§§^
Influenza (≥2 doses)***	53.8 (52.6 to 55.0)	58.1 (56.7 to 59.5)	4.3 (2.5 to 6.2)^§§^
**Combined 7-vaccine series^†††^**	68.4 (67.3 to 69.5)	70.5 (69.1 to 71.9)	2.1 (0.3 to 3.9)^§§^
**No vaccinations**	1.3 (1.1 to 1.5)	1.2 (1.0 to 1.4)	−0.1 (−0.3 to 0.2)

**Abbreviations:** CI = confidence interval; DTaP = diphtheria, tetanus toxoids, and acellular pertussis vaccine; HepA = hepatitis A vaccine; HepB = hepatitis B vaccine; Hib = *Haemophilus influenzae* type b conjugate vaccine; MMR = measles, mumps, and rubella vaccine; PCV = pneumococcal conjugate vaccine; VAR = varicella vaccine.

* Includes vaccinations received by age 24 months (before the day the child turns 24 months), except for the HepB birth dose, rotavirus vaccination, and ≥2 HepA doses by 35 months. For all vaccines except the HepB birth dose and rotavirus vaccination, the Kaplan-Meier method was used to estimate vaccination coverage to account for children whose vaccination history was ascertained before age 24 months (35 months for ≥2 HepA doses).

^†^ Data for the 2014 birth year are from survey years 2015, 2016, and 2017; data for the 2015 birth year are from survey years 2016, 2017, and 2018; data for the 2016 birth year are from survey years 2017, 2018, and 2019; data for the 2017 birth year are considered preliminary and come from survey years 2018 and 2019 (data from survey year 2020 are not yet available).

^§^ Includes children who might have been vaccinated with diphtheria and tetanus toxoids vaccine or diphtheria, tetanus toxoids, and pertussis vaccine.

^¶^ Includes children who might have been vaccinated with measles, mumps, rubella, and varicella combination vaccine.

** Hib primary series: receipt of ≥2 or ≥3 doses, depending on product type received; full series: primary series and booster dose, which includes receipt of ≥3 or ≥4 doses, depending on product type received.

^††^ One dose HepB administered from birth through age 3 days.

^§§^ Statistically significantly different from zero at p<0.05.

^¶¶^ Includes ≥2 doses of Rotarix monovalent rotavirus vaccine (RV1), or ≥3 doses of RotaTeq pentavalent rotavirus vaccine (RV5); if any dose in the series is RotaTeq or unknown, a 3-dose series was assumed. The maximum age for the final rotavirus dose is 8 months, 0 days.

*** Doses must be ≥24 days apart (4 weeks with a 4-day grace period); doses could have been received during two influenza seasons.

^†††^ The combined 7-vaccine series (4:3:1:3*:3:1:4) includes ≥4 doses of DTaP, ≥3 doses of poliovirus vaccine, ≥1 dose of measles-containing vaccine, the full series of Hib (≥3 or ≥4 doses, depending on product type), ≥3 doses of HepB, ≥1 dose of VAR, and ≥4 doses of PCV.

## Vaccination by Selected Sociodemographic Characteristics and Geographic Location

Coverage with all vaccines except the HepB birth dose was lower among uninsured children and those insured by any Medicaid plan (with or without another type of insurance) than among privately insured children (Table 2). Differences in coverage between uninsured children and those with private insurance ranged from 9.5 percentage points (≥3 HepB) to 33.9 percentage points (≥2 doses of influenza vaccine). Disparities between children insured by any Medicaid and those with private insurance tended to be smaller, ranging from 2.7 percentage points (≥1 VAR) to 20.3 percentage points (≥2 doses of influenza vaccine). The proportion of children who had received no vaccines was higher among uninsured (4.1%) and Medicaid-insured children (1.3%) than those privately insured (0.8%). Disparities in coverage were also observed by race/ethnicity (Supplementary Table 1, https://stacks.cdc.gov/view/cdc/95228), poverty level (Supplementary Table 2, https://stacks.cdc.gov/view/cdc/95260), and metropolitan statistical area (MSA)^§§^ status (Supplementary Table 2, https://stacks.cdc.gov/view/cdc/95260). The disparities, although smaller in magnitude than those associated with health insurance status, were present for nearly all vaccines based on poverty status but were much less consistent for race/ethnicity or MSA status. Estimated coverage varied widely by state/local area (Supplementary Table 3, https://stacks.cdc.gov/view/cdc/95261), most notably for ≥2 doses of influenza vaccine, with estimates ranging from 38.9% in Florida to 81.7% in Massachusetts (Figure).

**TABLE 2:** Estimated vaccination coverage by age 24 months* among children born during 2016–2017,^†^ by selected vaccines and doses and health insurance status^§^ — National Immunization Survey-Child, United States, 2017–2019

Vaccine/Dose	Health insurance status, % (95% CI)			
	Private only (referent)	Any Medicaid	Other insurance	Uninsured
	n = 13,659	n = 9,278	n = 2,226	n = 807
**DTaP^¶^**				
≥3 doses	95.9 (94.9–96.7)	91.3 (90.0–92.5)**	93.5 (91.5–95.1)**	84.7 (80.5–88.5)**
≥4 doses	86.0 (84.3–87.5)	76.6 (74.5–78.5)**	79.6 (75.6–83.2)**	65.6 (59.8–71.3)**
**Poliovirus (≥3 doses)**	95.0 (93.9–95.9)	90.1 (88.7–91.4)**	92.0 (89.7–93.8)**	82.7 (78.2–86.7)**
**MMR (≥1 dose)^††^**	92.8 (91.4–94.0)	89.4 (87.9–90.8)**	90.7 (88.3–92.8)	79.6 (74.9–84.0)**
**Hib^§§^**				
Primary series	94.6 (93.3–95.7)	90.4 (89.0–91.8)**	92.8 (90.7–94.6)**	82.9 (78.5–86.9)**
Full series	85.2 (83.5–86.7)	75.7 (73.6–77.7)**	81.3 (77.5–84.8)**	61.7 (56.0–67.5)**
**HepB**				
Birth dose^¶¶^	77.3 (75.6–78.9)	76.3 (74.2–78.3)	72.4 (67.5–76.8)	72.5 (67.1–77.3)
≥3 doses	93.2 (92.1–94.1)	90.1 (88.5–91.5)**	92.1 (90.0–94.0)	83.7 (79.4–87.5)**
**VAR (≥1 dose)^††^**	92.2 (90.8–93.4)	89.5 (88.0–90.9)**	87.9 (85.0–90.4)**	74.8 (69.3–80.1)**
**PCV**				
≥3 doses	94.2 (93.0–95.3)	89.8 (88.4–91.1)**	92.1 (89.9–93.9)	81.3 (76.8–85.4)**
≥4 doses	87.5 (86.0–89.0)	77.3 (75.3–79.3)**	81.1 (77.3–84.6)**	64.0 (58.0–70.0)**
**HepA**				
≥1 dose	88.0 (86.4–89.5)	84.7 (83.0–86.3)**	85.4 (82.5–88.1)	71.5 (65.9–76.9)**
≥2 doses (by age 35 mos)	80.5 (78.5–82.5)	75.7 (72.6–78.6)**	75.1 (70.3–79.6)**	49.2 (41.9–57.1)**
**Rotavirus** (by age 8 mos)***	84.6 (83.2–85.9)	67.5 (65.3–69.6)**	76.3 (72.7–79.6)**	55.7 (49.5–61.7)**
**Influenza (≥2 doses)^†††^**	69.6 (67.7–71.4)	49.3 (47.1–51.6)**	53.8 (48.7–59.1)**	35.7 (30.2–41.9)**
**Combined 7-vaccine series^§§§^**	76.9 (75.1–78.7)	65.7 (63.4–67.9)**	70.4 (65.8–74.8)**	50.6 (44.7–56.8)**
**No vaccinations**	0.8 (0.6–1.1)	1.3 (1.0–1.6)**	1.7 (1.0–2.7)	4.1 (2.7–5.9)**

**Abbreviations:** CI = confidence interval; DTaP = diphtheria, tetanus toxoids, and acellular pertussis vaccine; HepA = hepatitis A vaccine; HepB = hepatitis B vaccine; Hib = *Haemophilus influenzae* type b conjugate vaccine; MMR = measles, mumps, and rubella vaccine; PCV = pneumococcal conjugate vaccine; VAR = varicella vaccine.

* Includes vaccinations received by age 24 months (before the day the child turns 24 months), except for the HepB birth dose, rotavirus vaccination, and ≥2 HepA doses by age 35 months. For all vaccines except the HepB birth dose and rotavirus vaccination, the Kaplan-Meier method was used to estimate vaccination coverage to account for children whose vaccination history was ascertained before age 24 months (35 months for ≥2 HepA doses).

^†^ Data for the 2016 birth year are from survey years 2017, 2018, and 2019; data for the 2017 birth year are considered preliminary and come from survey years 2018 and 2019 (data from survey year 2020 are not yet available).

^§^ Children’s health insurance status was reported by parent or guardian. “Other insurance” includes the Children’s Health Insurance Program (CHIP), military insurance, coverage via the Indian Health Service, and any other type of health insurance not mentioned elsewhere.

^¶^ Includes children who might have been vaccinated with diphtheria and tetanus toxoids vaccine or diphtheria, tetanus toxoids, and pertussis vaccine.

** Statistically significant (p<0.05) difference compared with the referent group.

^††^ Includes children who might have been vaccinated with measles, mumps, rubella, and varicella combination vaccine.

^§§^ Hib primary series: receipt of ≥2 or ≥3 doses, depending on product type received; full series: primary series and booster dose, which includes receipt of ≥3 or ≥4 doses, depending on product type received.

^¶¶^ One dose HepB administered from birth through age 3 days.

*** Includes ≥2 doses of Rotarix monovalent rotavirus vaccine (RV1), or ≥3 doses of RotaTeq pentavalent rotavirus vaccine (RV5); if any dose in the series is RotaTeq or unknown, a 3-dose series was assumed. The maximum age for the final rotavirus dose is 8 months, 0 days.

^†††^ Doses must be ≥24 days apart (4 weeks with a 4-day grace period); doses could have been received during two influenza seasons. Children aged 6 months to 8 years should receive 2 doses separated by ≥4 weeks if they did not receive ≥2 doses during the previous flu season.

^§§§^ The combined 7-vaccine series (4:3:1:3*:3:1:4) includes ≥4 doses of DTaP, ≥3 doses of poliovirus vaccine, ≥1 dose of measles-containing vaccine, the full series of Hib (≥3 or ≥4 doses, depending on product type), ≥3 doses of HepB, ≥1 dose of VAR, and ≥4 doses of PCV.

**FIGURE:**
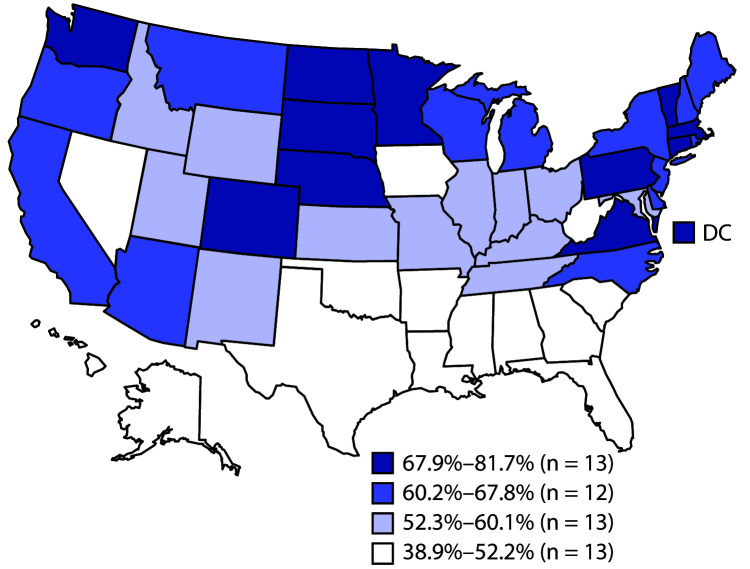
Estimated vaccination coverage with ≥2 doses of influenza vaccine* by age 24 months, among children born during 2016–2017^†^ — National Immunization Survey-Child, United States, 2017–2019 **Abbreviation:** DC = District of Columbia. * Doses must be ≥24 days apart (4 weeks with a 4-day grace period); doses could have been received during two influenza seasons. ^†^ Data from the 2016 birth year are from survey years 2017, 2018, and 2019; data for the 2017 birth year are considered preliminary and come from survey years 2018 and 2019 (data from survey year 2020 are not yet available).

## Discussion

For most ACIP-recommended childhood vaccines, coverage was stable by year of birth from 2011 to 2017.^¶¶^ The percentage of children who received no vaccinations ranged from 0.9% for those born in 2011 and 2017 to 1.5% for those born in 2016; the linear relationship between the prevalence of children receiving no vaccinations and birth year was not statistically significant.*** More recent increases have been observed for ≥2 doses of influenza vaccine, the HepB birth dose, completion of the rotavirus vaccination series, ≥1 dose of HepA, and the combined 7-vaccine series. However, not all children have benefited from the high and increasing national-level coverage. Coverage among uninsured children and those insured by Medicaid is lower than that among privately insured children. The lowest coverage and largest insurance-related disparities were associated with ≥2 doses of influenza vaccine; increasing influenza vaccination coverage is particularly important this season, given the likely cocirculation of influenza virus and SARS-CoV-2, the virus that causes COVID-19.

Children aged 6–59 months are at increased risk for severe illness and complications from influenza and for influenza-related outpatient, emergency department, or hospital visits (*2*). Most children are recommended to receive 3 doses of influenza vaccine by age 24 months, depending on their month of birth and the months considered as the seasonal influenza vaccination period (*2*). Thus, the percentage of children fully vaccinated by age 24 months per ACIP recommendations is lower than the estimates for receipt of ≥2 influenza vaccine doses in this report, which are based on criteria from the Healthcare Effectiveness Data and Information Set (HEDIS).^†††^ Current efforts to increase influenza vaccination coverage are especially important, given that SARS-CoV-2 and influenza virus are likely to be circulating in the population simultaneously during the fall and winter of 2020–21. Both viruses are associated with significant morbidity and mortality, and together they could impose considerable strain on the public health and medical systems in the United States (*3*,*4*).

Coverage with influenza and most other vaccines was lower for children with Medicaid or no health insurance. The Vaccines for Children (VFC) program^§§§^ provides recommended vaccines at no cost to children aged ≤18 years who are Medicaid-eligible, uninsured, American Indian/Alaska Native, or insured by health plans that do not fully cover all routine immunization; however, parents of eligible children might be unaware of VFC or might face difficulty accessing vaccination services. Increased awareness of the program and assistance locating VFC providers could facilitate improved vaccination coverage among eligible children. Observed coverage was also lower among children living in poverty. Although this could indicate challenges accessing VFC, for which many of these children likely qualify, lower family income has also been associated with more parental vaccine hesitancy (*5*). Strategies for responding to vaccine hesitancy and other barriers to vaccination are described in a framework newly developed by CDC and its partners called Vaccinate with Confidence (*6*), which outlines activities designed to increase vaccination coverage by helping to protect communities, empower families, and stop vaccination-related myths.

The findings in this report are subject to at least two limitations. First, the low response rate and exclusion of phoneless and landline-only households creates the possibility for bias if study participants are not representative of U.S. children of the corresponding age. Second, coverage could be underestimated as a result of an incomplete list of vaccination providers identified by parents or providers not returning the vaccination history survey. A recent assessment of total survey error^¶¶¶^ has shown that NIS-Child estimates might slightly underestimate true coverage for MMR and ≥4 DTaP, and by as much as nine percentage points for the combined 7-vaccine series. Evidence for a change in survey accuracy from 2018 to 2019 was not apparent.**** Estimates of coverage with ≥2 influenza vaccine doses by age 24 months might differ from other CDC estimates that are specific to each influenza season or based on parent report of their child’s vaccination status (*7*).

By the early spring of 2020, the COVID-19 pandemic was rapidly expanding in the United States, and as the number of cases increased over the subsequent weeks and months, state and local governments increasingly imposed stay-at-home orders in an effort to slow the spread of disease.^††††^ Although CDC continued to emphasize the importance of well child exams and immunization during the pandemic, disruptions occurred in nearly all parts of society, including routine medical care such as vaccination (*8*). Extra effort to ensure that children continue receiving life-saving vaccines, especially uninsured children and those insured by Medicaid, is critical. Many providers’ ability to deliver routinely recommended childhood vaccines has likely recovered following the initial impact of the pandemic (*9*,*10*). Health care and public health authorities can communicate with families about how vaccinations can be provided safely during the pandemic, remind parents of vaccinations that are due or overdue for their children, and administer all recommended vaccinations to children during clinic visits. Providers should use every opportunity to safely administer recommended vaccines to children during the COVID-19 pandemic, with particular attention to influenza vaccination during fall and winter.^§§§§^

**Summary**

**What is already known about this topic?**

The National Immunization Survey-Child monitors coverage with vaccines recommended for children age <24 months to protect against 14 potentially serious illnesses.

**What is added by this report?**

National coverage with many recommended vaccines has remained high and stable, with recent increases for several vaccines for children born during 2016–2017 compared with those born during 2014–2015. Large coverage disparities by health insurance and poverty status persist.

**What are the implications for public health practice?**

The COVID-19 pandemic has disrupted routine medical care. Extra effort will be required to achieve and maintain high levels of coverage with recommended childhood vaccinations. This is especially important for seasonal influenza vaccination to mitigate the effect of cocirculation of two serious respiratory viruses.

## Footnotes

* https://www.who.int/health-topics/vaccines-and-immunization#tab=tab_1.

† Estimates for states, selected local areas, and the territories of Guam and Puerto Rico are available online at https://www.cdc.gov/vaccines/imz-managers/coverage/childvaxview/data-reports/index.html. Certain local areas that receive federal Section 317 immunization funds are sampled separately and included in the NIS-Child sample every year (Chicago, Illinois; New York, New York; Philadelphia County, Pennsylvania; Bexar County, Texas; and Houston, Texas). Other local areas in Texas were sampled in some survey years from 2017–2019 and not others, including El Paso County (survey years 2017 and 2019); Dallas County (survey years 2017 and 2019); Hidalgo County (survey year 2018); and Tarrant County (survey year 2018). Data collection in Puerto Rico was suspended during 2017 because of the severity of the hurricane season and did not occur at all in 2018. Therefore, estimates for Puerto Rico are based on partial data from 2017 and data collected in the full 2019 survey year. National estimates in this report exclude all territories.

§ The NIS-Child used a landline-only sampling frame from 1995 through 2010. From 2011 through 2017, the survey was conducted using a dual-frame design, with both mobile and landline sampling frames included. In 2018, the NIS-Child returned to a single-frame design, with all interviews conducted by mobile telephone.

¶ Children with at least one vaccination reported by a provider and those who had received no vaccinations were considered to have adequate provider data. “No vaccinations” indicates that the vaccination status is known because the parent or guardian indicated that there were no vaccinations, and the providers returned no immunization history forms or returned them indicating that no vaccinations had been given.

** The Council of American Survey Research Organizations (CASRO) household response rate is calculated as the product of the resolution rate (percentage of the total telephone numbers called that were classified as nonworking, nonresidential, or residential), screening completion rate (percentage of known households that were successfully screened for the presence of age-eligible children), and the interview completion rate (percentage of households with one or more age-eligible children that completed the household survey). The CASRO household response rate is equivalent to the American Association for Public Opinion Research type 3 response rate http://www.aapor.org/AAPOR_Main/media/publications/Standard-Definitions20169theditionfinal.pdf. CASRO response rates and the proportions of children with household interviews that had adequate provider data for survey years 2015–2018 are available at https://www.cdc.gov/vaccines/imz-managers/nis/downloads/NIS-PUF18-DUG.pdf.

†† The combined 7-vaccine series (4:3:1:3*:3:1:4) includes ≥4 doses of diphtheria and tetanus toxoids and acellular pertussis vaccine; ≥3 doses of poliovirus vaccine; ≥1 dose of measles-containing vaccine; ≥3 or ≥4 doses (depending upon product type) of *Haemophilus influenzae* type b conjugate vaccine; ≥3 doses of hepatitis B vaccine; ≥1 dose of varicella vaccine; and ≥4 doses of pneumococcal conjugate vaccine.

§§ MSA status was determined based on household reported city and county of residence and was grouped into three categories: MSA principal city, MSA nonprincipal city, and non-MSA. MSAs and principal cities were as defined by the U.S. Census Bureau (https://www.census.gov/programs-surveys/metro-micro.html). Non-MSA areas include urban populations not located within an MSA as well as completely rural areas.

¶¶ https://www.cdc.gov/vaccines/imz-managers/coverage/childvaxview/pubs-presentations/NIS-child-vac-coverage-2016-2017-tables.html#supp-figure-01.

*** https://www.cdc.gov/vaccines/imz-managers/coverage/childvaxview/pubs-presentations/NIS-child-vac-coverage-2016-2017-tables.html#supp-figure-02.

††† https://www.ncqa.org/hedis/measures/childhood-immunization-status/.

§§§ https://www.cdc.gov/vaccines/programs/vfc/.

¶¶¶ https://www.cdc.gov/vaccines/imz-managers/nis/downloads/NIS-PUF18-DUG.pdf.

**** https://www.cdc.gov/vaccines/imz-managers/coverage/childvaxview/pubs-presentations/NIS-child-vac-coverage-2016-2017-tables.html#supp-table-01.

†††† https://www.finra.org/rules-guidance/key-topics/covid-19/shelter-in-place.

§§§§
https://www.cdc.gov/vaccines/pandemic-guidance/index.html .
